# Electromyographic amplitude versus torque relationships are different in young versus postmenopausal females and are related to muscle mass after controlling for bodyweight.

**DOI:** 10.1007/s00421-020-04532-0

**Published:** 2020-10-29

**Authors:** Nile F. Banks, Emily M. Rogers, Nathaniel D.M. Jenkins

**Affiliations:** 1 Applied Neuromuscular Physiology Laboratory, Oklahoma State University, Stillwater, OK; 2 Laboratory for Applied Nutrition and Exercise Science, Oklahoma State University, Stillwater, OK

**Keywords:** electromyography, skeletal muscle, aging, menopause, muscle function

## Abstract

**Purpose:**

To examine differences in the electromyographic vs torque (EMG-T) relationship, as well as muscle strength and indicators of muscle mass and quality between young versus postmenopausal females, and explore whether the potential differences in the EMG-T relationships could be explained by differences in muscle mass.

**Methods:**

Thirty young (age = 20.7 ± 2.8 y) and 30 postmenopausal (age = 56.3 ± 4.7 y) females completed maximal isometric strength testing (MVIT) and isometric ramp contractions at 40% and 70% MVIT, during which electromyographic signals were collected to quantify the slopes (Slope_40_; Slope_70_) and intercepts (Intercept_40_; Intercept_70_) of the EMG-T relationships. Muscle mass and quality measurements were also completed.

**Results:**

Postmenopausal females exhibited lower skeletal muscle mass (−2.3 ± 1.5 kg), fat free mass index (−1.1 ± 0.7 kg·m^−2^), MVIT (−17.1 ± 16.3 Nm), phase angle (−0.5 ± 0.0°), muscle cross sectional area (−5.5 ± 1.1 cm^2^), muscle quality (−0.1±0.0 a.u), Slope_40_ (−0.0003 ± 0.0002 mV·%MVIT^−1^), Slope_70_ (−0.0003 ± 0.0002 mV·%MVIT^−1^), and had a higher echo intensity (+9.8 ± 2.8 a.u), Intercept_40_ (+0.001 ± 0.001 mV), and Intercept_70_ (+0.004 ± 0.003 mV) (p ≤ 0.001 – 0.04) than the young females. The EMG-T relationship variables were correlated with both muscle mass and quality after controlling for bodyweight. When controlling for muscle mass and bodyweight, group differences in the slopes of the EMG-T relationship and muscle strength were eliminated.

**Conclusion:**

Muscle mass and quality are primary contributors to the decrements in neuromuscular function observed in postmenopausal versus young females, and the preservation of muscle mass should be prioritized in the years leading up to, during, and immediately after menopause.

## Introduction

Aging is associated with a general loss of functional ability and independence, and it is of primary importance to understand the contributing factors to be able to better slow the rate of decline in an increasingly growing population of older adults (Milanovic et al. 2013). Skeletal muscle health and function appear to be primary contributing factors (Tieland et al. 2018), and age-related decreases in skeletal muscle mass (SMM; i.e., sarcopenia) and skeletal muscle quality have been implicated as major contributors to decreased functional ability (Cramer et al. 2017; Jenkins et al. 2015; Keller and Engelhardt 2013; Phillips et al. 1993). While these impairments have been shown to decline with age independent of sex (Lang et al. 2010), the rate of decline appears to increase rather dramatically at or near the time of menopause in females. For example, Aloia et al. (1991) reported an accelerated loss of total body potassium, a marker of lean body mass, at menopause. Further, Phillips et al. (1993) reported a dramatic reduction in specific muscle force at the time of menopause. Consequently, a higher incidence of sarcopenia has been reported among females than males between the ages of 60 and 70 y (Baumgartner et al. 1998; Kirchengast and Huber 2009; Shafiee et al. 2017). Despite these observations, however, there is a relative lack of research regarding the influence of menopause on neuromuscular function in females.

Central nervous system limitations are another major factor contributing to age-related decreases in force production capabilities. Specifically, deficits in voluntary activation, or the ability to fully activate the motor unit (MU) pool and drive active MUs to maximal discharge rates (Tieland et al. 2018; Jenkins et al. 2017), are thought to contribute to the observation of lower electromyographic signal (EMG) amplitudes at a variety of torque requirements in older versus younger adults (Clark et al. 2010; Rozand et al. 2020). The relationship between muscle activity measured through surface electromyography (sEMG) and isometric torque, or the EMG versus torque (EMG-T) relationship, provides complex information regarding neural and muscular contributions to force production. Consequently, it has been suggested that the EMG-T relationship may give insight into the MU activation strategies or muscle activation strategies used to produce force during progressive, non-fatiguing contractions (Herda et al. 2011; Paquin and Power 2018). Indeed, Herda et al. (2011) reported that the EMG-T relationship distinguished between young adults with high- (> 90%) versus moderate- (< 90%) voluntary activation capabilities. Thus, the EMG-T relationship may be a useful tool to examine neuromuscular impairments associated with age and/or menopause.

One issue with inferring differences in neuromuscular control between subjects utilizing the EMG-T relationship is the known influence of peripheral factors such as muscle geometry on the EMG signal. In fact, Martinez-Valdes et al. (2018) recently reported that differences in EMG amplitude between the vastus lateralis (VL) and vastus medialis muscles are primarily explained by differences in motor unit action potential (MUAP) sizes (r = 0.73 – 0.85), which likely reflect muscle fiber geometries. Interestingly, Sterczala et al. (2018) reported smaller MUAP amplitudes of high-threshold MUs in older than younger individuals and suggested that this was caused by atrophy of the fibers that comprised the higher-threshold MUs. Therefore, due to the influence of peripheral factors such as muscle fiber sizes on the amplitude of the EMG signal, it is plausible that any age- or menopause-related differences in the EMG-T relationship may be influenced by muscle atrophy, and that older adults would have less steep slopes compared to their younger counterparts due to preferential atrophy of fibers comprising higher threshold MUs.

The primary purposes of this study were (a) to examine differences in the EMG-T relationship, as well as muscle strength and indicators of muscle mass and quality between young versus postmenopausal females, and (b) to explore whether the potential differences in the EMG-T relationships could be explained by differences in muscle mass in our sample.

### Participants

Thirty healthy young (mean ± SD; age = 20.7 ± 2.8 y; weight = 70.3 ± 13.9 kg; height = 163.8 ± 7.4 cm) and thirty healthy postmenopausal (56.3 ± 4.7 y; 70.1 ± 10.3 kg; 162.3 ± 6.5 cm; years post-menopause = 6.6 ± 3.9 y) females volunteered for, and completed this study. In order to be eligible for the study, each participant must have been untrained and reported completing < 150 min·week^−1^ of moderate or < 75 min·week^−1^ of vigorous intensity exercise (per the American Heart Association guidelines (Piercy and Troiano 2018)), and have been a female between the ages of 18–29 or 45–65 y, free of any cardiovascular, metabolic, or neuromuscular disease, and could not have been currently taking any medications known to alter energy metabolism as determined by a health history questionnaire. In order to be eligible for the postmenopausal group, participants must have gone without a menstrual cycle for more than one year. No participants in the postmenopausal group had, or were undergoing hormone replacement therapy prior to or upon enrollment in the study. This study was approved by and conducted in accordance with the guidelines and regulations of the Oklahoma State University’s Institutional Review Board (IRB Approval #s: ED-17–157 and ED-18–101).

### Experimental Design

Each participant visited the laboratory on three separate occasions. During visit 1, participants completed the informed consent, health and exercise history questionnaire, and had their height and weight measured. Following an overnight fast, participants returned for visit two, and their body composition was assessed using a medical body composition analyzer. During visit 3, ultrasound-based assessments of VL muscle size and echo intensity (EI) were performed. After the ultrasound measurements, the participants completed maximal voluntary isometric leg-extension tests before completing submaximal isometric leg-extension tests, during which they traced a ramped torque template. During the submaximal contractions, EMG signals were collected from the VL to quantify the EMG-T relationship for each participant. To avoid any influence of the menstrual cycle in the young females, all testing was conducted during the last half of the follicular phase for these participants.

### Isometric Strength Testing

Subjects were seated on an isokinetic dynamometer (Biodex System 4; Biodex Medical Systems, Inc. Shirley, NY, USA) with the trunk and pelvis secured with straps and the lateral epicondyle aligned with the dynamometer head. All isometric testing was performed with a 90° knee joint angle and the lever arm pad positioned 3–4 cm proximal to the medial malleolus. Once secured in the dynamometer, participants completed three, 3-s warm-up leg extension muscle actions at intensities of 25, 50, and 75% of their perceived effort with 30-s of rest between each set of muscle actions. Following a 60-s rest period, the participants performed two, 5-s maximal voluntary contractions (MVIC) separated by at least 60-s of rest. If the second MVIC attempt rendered a higher peak value compared to the first attempt, the participant was given another 60-s rest period and additional attempts were completed until a true maximum was achieved. All torque signals were displayed in real-time on an external computer monitor for visual feedback and strong verbal encouragement was provided. The maximal voluntary isometric torque (MVIT) output (Nm) for each subject achieved in a 1-s window during either MVIC and was used to scale the subsequent submaximal, trapezoidal ramp contractions. Following MVIC testing, participants performed two trapezoidal ramp contractions at 40 and 70% MVIT by tracing a ramped torque trajectory displayed on an external computer monitor. The trajectories increased linearly at a rate of 10% MVIT·s^−1^ to 40 or 70% MVIT, were held for 10-s, and then decreased linearly at a rate of 10% MVIT·s^−1^ until returning to baseline. If the participant was unable to accurately trace a torque trajectory, they were provided a standard rest period and the tracing was repeated until two accurate trapezoidal ramp contractions were executed.

### Electromyography

Parallel-bar, bipolar, sEMG sensors with an interelectrode distance of 10 mm (Delsys DE-2.1, Delsys, Inc. Natick, MA, USA) were placed on the VL in order to quantify EMG amplitude during the trapezoidal ramp contractions. Prior to sensor placement, the surface of the skin was prepared by shaving and removing dead skin via careful abrasion, adhesive taping, and cleansing with alcohol. For the VL, the center of the bipolar electrode was placed at 66% of the distance between the anterior superior iliac spine and the lateral superior border of the patella (Hermens et al. 2000) and was oriented parallel to the angle of pennation of the muscle fibers (~20% (Fukumoto et al. 2012)). A reference electrode (UltraStim USX2000, Axelgaard Manufacturing Co., Ltd., Fallbrook, CA, USA) was placed on the spinous process of the C7.

### Signal Processing

The EMG and torque signals were sampled simultaneously at 2 kHz using a Delsys Bagnoli Desktop data acquisition system (Delsys, Inc., Natick, MA, USA), recorded to a desktop computer, and processed offline using custom written Labview software (v.12.0, National Instruments, Austin, TX, USA). The EMG signals were amplified using the built-in sensor amplifier using a gain of 10 VN ± 1%, a common mode rejection ratio of −92 dB, and an input impedance of >1015Ω//0.2pF. The EMG signals were then zero-meaned and bandpass filtered from 10 – 499 Hz using a zero-phase shift, 4^th^-order Butterworth filter. The torque signals were low-pass filtered at 20 Hz using a zero-phase shift, 4^th^-order Butterworth filter.

**Figure 1.**
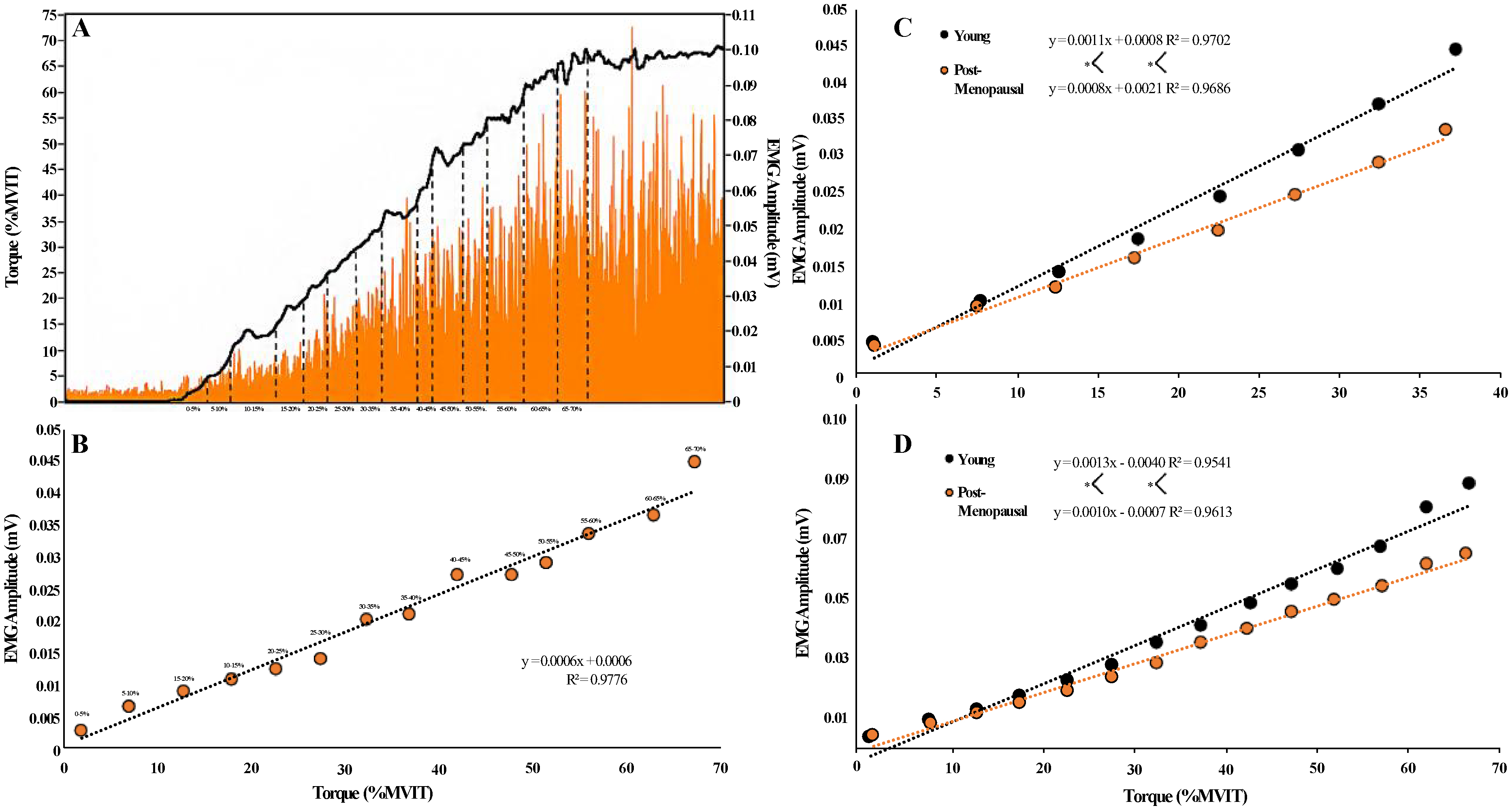
**A**: The filtered and rectified *electromyographic (*EMG*)* and filtered torque signal collected during a trapezoidal tracing at 70% of maximal voluntary isometric torque (MVIT) for one participant. The vertical dashed lines illustrate the bins in which EMG *root mean square amplitude (*RMS*)* and average torque were calculated during the linearly increasing torque segment. **B**: Shows the EMG RMS versus average torque values from the corresponding 5% MVIC bins for the same participant for whom the signals are displayed in figure A, as well as the line and equation characterizing the EMG versus torque relationship *for this subject*. **C**: The mean EMG versus average torque values during the 40% trapezoidal tracing with the composite regression equation *in the young and postmenopausal females*. **D**: The mean EMG versus average torque values during the ramp contraction at 70% trapezoidal tracing with the composite regression equation *in the young and postmenopausal females*. In C and D, the black circles and lines represent the young females, whereas the orange-filled circles and orange lines represent the postmenopausal.

During the MVICs, MVIT and EMG amplitude values were calculated from the 1,000 ms epoch corresponding to the highest average torque value that occurred during the MVIC plateau. For each 5% increment of torque during the increasing portion of the submaximal ramp contractions at 40 and 70% MVIC, the average torque achieved and the associated EMG amplitude values were calculated (Figure 1). The EMG amplitude values were expressed as the root mean square value in mV. Subsequently, the EMG-T relationship was determined for each subject during the submaximal ramp contractions by regressing EMG amplitude against average torque, which provided a slope (mV·MVIT%^−1^) and y-intercept (mV) value for each subject during both 40 (Slope_40_; Intercept_40_) and 70% (Slope_70_; Intercept_70_) submaximal ramp contractions.

### Body Composition

Participants were asked to arrive to the laboratory euhydrated prior to body composition analysis. Height and weight were measured using an eye level beam physician scale (Physician Scale 439; Detecto, Webb City, MO, US) and a calibrated platform scale (ADAM CPWplus 150 M, Oxford, CT, USA), respectively. Following a 10-h overnight fast, SMM (kg), fat free mass index (FFMI; FM (kg) / Height (m)^2^), and phase angle (PA; degree) were assessed using 8-point bioelectrical impedance analysis (BIA; SECA mBCA 514, Hamburg, DE) as described in detail previously (Bosy-Westphal et al. 2013). During BIA, resistance (R) and reactance (Xc) were measured in ohms (Ω), and PA (°) was calculated using the following formula:

$$PA=tan^{-1}\left(\frac{Xc}{R}\right)\times \left(\frac{180}{\pi }\right)$$

(Barbosa-Silva et al. 2005).

### Skeletal Muscle Ultrasound

During all ultrasound testing, the participant’s right leg was examined while in the supine position with a pillow placed behind the knees and the leg braced in a custom-made foot mold to prevent rotation of the femur. Ultrasound images were obtained using a brightness mode (B-mode) ultrasound device (General Electric LOGIQ S8, Wauwatosa, WI) and a multi-frequency linear-array probe (Model ML6–15-D 4–15 MHz, 50-mm field of view). A generous amount of transmission gel was applied to the skin and the ultrasound probe to maximize acoustic coupling and to reduce near-field artifacts, and minimal pressure was applied to the probe during all measurements to limit compression of the muscle. The frequency was set at 12 MHz, the image gain at 50 dB, the dynamic range at 72 dB, and the image depth was set between 5–9 cm. Three panoramic ultrasound images of the VL were taken at 50% of the distance from the anterior superior iliac spine to the proximal border of the patella. The muscle cross sectional area (mCSA) of the VL was then analyzed using image analysis software (ImageJ, version 1.50i) by selecting a region of interest including as much muscle as possible without including surrounding fascia using the built-in polygon function. VL EI was quantified as arbitrary unit (a.u.) values between 0 (black) and 255 (white) by gray-scale analysis of each image using the histogram function of ImageJ from the same region of interest as mCSA. Muscle quality (MQ) was then quantified by normalizing VL mCSA to VL EI (cm^2^/a.u.).

### Nutritional Status

Participants were asked to record and turn in dietary logs containing all food and calorie-containing beverages consumed during two week days, and one weekend day. Participants recorded food immediately after consumption, and provided information regarding the method of preparation and serving sizes. Calories (kcals) and protein (g) intake were then calculated by entering all foods and drinks consumed into ESHA’s Food Processor® Nutrition Analysis Software (https://www.esha.com, ESHA Research, Oak Brook, Il, USA) (Bazzano et al. 2002; Liu et al. 2013).

### Statistical Analyses

Independent-sample t-tests were used to examine age-related differences in the dependent variables, which included age, height, weight, caloric and protein intakes, SMM, FFMI, MVIT, PA, mCSA, EI, MQ, Slope_40_, Intercept_40_, Slope_70_, and Intercept_70_. The assumption of homogeneity was assessed for all t-tests using Levene’s Test of Equality of Variances. If violated, the degrees of freedom were adjusted using the Welch-Satterthwaite method. Normality was also assessed using the Shapiro-Wilks test. Any non-normally distributed variables were analyzed using Mann-Whitney tests. Pearson’s partial correlation coefficients were calculated to examine the relationship among all of the dependent variables after adjusting for bodyweight, because bodyweight has been shown to exert a substantial influence on neuromuscular function outcomes (Folland et al. 2008; Oba et al. 2014; Weir et al. 1999) and it displayed collinearity with the other neuromuscular function variables in this study (i.e. MVIT, SMM, mCSA, MQ, PA, and EMG-T slopes and intercepts). Age was not included in the correlation analysis, because it was non-continuous due to the recruitment of two distinct age groups. Strengths of association were deemed small, moderate, or strong when the coeffeicient values were between 0.1 – 0.3, 0.3 – 0.5, and > 0.5, respectively.

Based on our initial correlation analyses, we then conducted six separate analyses of covariance (ANCOVAs) to explore whether potential between group differences in the EMG-T relationship and MVIT could be explained by differences in indicators of muscle mass or muscle quality and bodyweight. For these analyses, the dependent variables of interest were MVIT, Slope_40_, and Slope_70_, the independent variable was group (i.e., young vs. postmenopausal), and the covariates were bodyweight and SMM or bodyweight and MQ. All statistical analyses were performed using GraphPad Prism 8 (GraphPad Software Inc, San Diego, CA) and IBM SPSS Statistics (v. 26, IBM Corp, Armonk, NY). Unless otherwise reported, all mean differences are reported as the mean or median ± 95% confidence interval difference. The a priori type I error rate was set at 0.05.

## Results

All data were normally distributed except for age (p < 0.001), height (p = 0.032), weight (p = 0.002), caloric intake (p = 0.025), protein intake (p = 0.029), MQ (p = 0.008), Slope_40_ (p = 0.046), Slope_70_ (p < 0.001), and Intercept_70_ (p < 0.001), and the homogeneity of variances assumption was met for all variables except for age (p = 0.01) and Intercept_40_ (p = 0.046). Table 1 includes age-related differences among the dependent variables. In summary, the postmenopausal females had lower SMM (−2.3 ± 1.5 kg; p = 0.004), FFMI (−1.1 ± 0.7 kg/m^2^; p = 0.002), MVIT (−17.1 ± 16.3 Nm; p = 0.04), PA (−0.5 ± 0.0°; p < 0.001), mCSA (−5.5 ± 1.1 cm^2^; p < 0.001), MQ (−0.1 ± 0.0 a.u.; p <0.001), Slope_40_ (−0.0003 ± 0.0002 mV·%MVIT^−1^; p = 0.008), and Slope_70_ (−0.0003 ± 0.0002 mV·%MVIT^−1^; p = 0.005) and had greater EI (+9.8 ± 2.8 a.u.; p = 0.001), Intercept_40_ (+0.0013 ± 0.0011 mV; p = 0.02), and Intercept_70_ (+0.0033 ± 0.0024 mV; p < 0.001) compared to the young females (Table 1). The relationships among the dependent variables after partialling out bodyweight are included in Table 2.

The ANCOVA analyses indicated that the age-related differences for Slope_40_ and Slope_70_ were both eliminated (both p ≥ 0.70) when co-varying for bodyweight and SMM, and both bodyweight (p ≤ 0.008; n^2^_p_ ≥ 0.12) and SMM (p ≤ 0.001; n^2^_p_ ≥ 0.22) were significant adjusters of the means for both Slope_40_ and Slope_70_. Similarly, when co-varying for bodyweight and MQ, the age-related differences for Slope_40_ and Slope_70_ were eliminated (both p ≥ 0.27), although while MQ was not a significant adjuster of the means (p ≥ 0.13; n^2^_p_ ≤ 0.04), bodyweight was (p ≤ 0.002; n^2^_p_ ≥ 0.16).

For MVIT, the ANCOVA analyses indicated that the age-related difference was significant adjuster of the means (p < 0.001; n^2^_p_ = 0.21), bodyweight was not (p = 0.36; n^2^_p_ = 0.02). Finally, when controlling for bodyweight and MQ, the age-related difference in MVIT was also eliminated (p = 0.84), and both MQ (p = 0.002; n^2^_p_ = 0.16) and bodyweight (p = 0.01; n^2^_p_ = 0.11) were significant adjusters of the mean.

## Discussion

The primary purposes of this study were (a) to examine differences in the EMG-T relationship, as well as muscle strength and indicators of muscle mass and quality between young versus postmenopausal females, and (b) to explore whether the potential differences in the EMG-T relationships could be explained by differences in muscle mass in our sample. The results of this study indicated that the young females exhibited steeper slopes and lower y-intercepts characterizing the EMG-T relationship during ramp contractions at both 40 and 70% of MVIT when compared to their postmenopausal female counterparts. We also observed differences in MVIT, SMM, FFMI, PA, mCSA, EI, and MQ among the young versus postmenopausal females. After controlling for bodyweight, measurements of SMM, MVIT, PA, mCSA, and EI were consistently related to the EMT-T slopes and intercepts. Additionally, the differences among the neuromuscular function indices (i.e., slopes of EMG-T relationship and MVIT) were eliminated after accounting for SMM or MQ and bodyweight, although SMM seems to be a more consistent, significant adjuster than MQ. Therefore, it appears that the observed differences in neuromuscular function among the young versus postmenopausal females in this study may primarily be driven by age-related differences in skeletal muscle mass.

The postmenopausal females in the present study exhibited lower Slope_40_ and Slope_70_, but greater Intercept_40_ and Intercept_70_ (Figure 1C and D). Thus, while the postmenopausal females displayed greater EMG amplitudes at very low torques, the young females displayed a more progressive increase in EMG amplitude across the torque spectrum. Moreover, the young females were 17% stronger compared to the postmenopausal females. Though the loss of SMM is major consequence of aging, there are other factors needed to fully explain age-associated impairments in muscle strength, such as decreases in skeletal muscle quality and intrinsic force generation capacity, as well as neural decrements that influence muscle activation (Cardozo et al. 2013; Clark and Fielding 2012). Interestingly, the EMG-T relationship has been shown to be sensitive to differences in voluntary activation capacity in young adults (Herda et al. 2011). Herda et al. (2011) observed greater increases in EMG amplitude from 30 – 90% MVIT in participants able to achieve > 90% voluntary activation than in those who achieved < 90% voluntary activation. In a recent systematic review, Rozand et al. (2020) reported a modest reduction of voluntary activation in the knee extensors of older versus younger adults. Therefore, it is possible that an age-associated reduction in voluntary activation contributed to the age-related differences observed for the slopes and intercepts of the EMG-T relationship in the present study. However, this is highly speculative and, as we discuss further below, it is extremely likely that the age-related differences in the EMG-T relationship were heavily influenced by differences in muscle fiber size.

While aging is generally characterized by a progressive decrease in SMM, a few studies indicate that menopause may cause an acceleration in this decline. For example, Greendale et al. (2019) reported a 0.2% decline of SMM per year during the menopause transition period and Aloia et al. (1991) reported an accelerated loss of total body potassium (a marker of lean body mass) after menopause and that the rate of loss was greatest for those ≤ 3 y (−1.3%) when compared to those > 6 y (−0.5%) post-menopause. In the present study, the postmenopausal females, who were ~56 y of age and ~6.6 y post-menopause, displayed 12% lower total SMM and 32% lower VL mCSA than the young females. Thus, our cross-sectional data, in combination with the data presented by Aloia et al. (1991), suggest that postmenopausal females experience a significant increase in the rate of SMM loss in the years immediately following menopause, causing large decreases in SMM by the middle of their sixth decade. Indeed, higher sarcopenia prevalence has generally been reported among females than in males between the ages of 60 and 70 y (Baumgartner et al. 1998; Kirchengast and Huber 2009; Shafiee et al. 2017).

The age-related loss of skeletal muscle mass, or sarcopenia, is characterized by progressive atrophy and loss of both type I and type II muscle fibers, although several studies have suggested that these changes appear to be accelerated for the latter (Larsson et al. 1978; Brooke and Kaiser 1970; Verdijk et al. 2007). This decrease in fiber size and function has also been suggested as a primary driver of age-related decreases in muscle strength (Cramer et al. 2017; Jenkins et al. 2015). Phillips et al. (1993) reported a drastic drop off in specific strength (strength/CSA) in females at the time of menopause that was prevented in individuals on hormone replacement therapy, and also reported strong correlations between strength and CSA. It has also been proposed that age-related decreases in muscle quality also contribute to age-related decreases in neuromuscular function. EI and PA have been suggested as surrogate measurements of muscle quality and cellular health, respectively (Garlini et al. 2019; Reimers et al. 1993). Indeed, EI has been shown to be strongly correlated with MRI-based assessments of intramuscular fat (Young et al. 2015), is inversely associated and is a significant independent predictor of maximal strength in older adult males (Cadore et al. 2012; Watanabe et al. 2013) and females (Fukumoto et al. 2012), and has been shown to increase with age (Magrini et al. 2018). Similarly, PA is thought to be associated with cellular health, has been used as a prognostic indicator in various health conditions (Garlini et al. 2019) and, accordingly, has been shown to decline with age (Barbosa-Silva et al. 2005; Dittmar 2003; Norman et al. 2015). In addition to EI and PA, we also normalized VL mCSA to EI as a more wholistic indicator of MQ. In the present study, EI was 13% greater, while PA and MQ were 10% and 45% lower in the postmenopausal versus young females, respectively. Thus, our data suggest that in addition to significant decreases in SMM, there are likely decreases in cellular health and/or muscle quality that may be contributing to the age-related decreases in neuromuscular function observed in the postmenopausal versus young females in this study.

In addition to the age-comparisons, we explored the relationships among muscle mass and quality and indices of neuromuscular function. After removing the influence of bodyweight, which has been shown to exert a significant influence on neuromuscular measurements (Folland et al. 2008; Oba et al. 2014; Weir et al. 1999) and indeed displayed collinearity with MVIT, SMM, mCSA, MQ, PA, and the EMG-T variables in the present investigation, both our indicators of muscle mass (i.e., SMM and mCSA) and quality (i.e., EI, PA, MQ) were consistently, moderately related to the neuromuscular function indices (i.e., MVIT, Slope_40_/Slope_70_, Intercept_40_/Intercept_70_). In addition to the aforementioned possibility that the age-related differences in MVIT and the EMG-T slopes and intercepts were caused by age-associated reductions in voluntary activation, it is also highly plausible and, in our opinion, more likely that differences in muscle mass and quality are responsible. Indeed, EMG amplitude is influenced by muscle architecture (Lowery et al. 2004). It has also been reported (Martinez-Valdes et al. 2018) that the difference in the absolute EMG amplitudes obtained at 70% MVIC between the vastus medialis versus VL are largely explained (57% of variability) by MUAP amplitudes, which have been proposed as a non-invasive indicator of muscle fiber size (Pope et al. 2016). Furthermore, in females, both Miller et al. (2013) and Trappe et al. (2003) have reported significant age-related decreases in myosin heavy chain IIa fiber size, specifically. Therefore, the observed relationships among muscle size, quality, and the EMG-T slopes in the present study, together with these previous studies, may indicate that the observed differences in the EMG-T relationship and MVIT are heavily influenced by age-related differences in the peripheral properties of skeletal muscle. To further explore this possibility, we also examined whether controlling for bodyweight and SMM or for bodyweight and MQ would eliminate the age-related differences in MVIT and slopes of the EMG-T relationships. Indeed, when accounting for these variables, the differences were eliminated, which provides additional evidence that the differences in neuromuscular function, as assessed with MVIT and the EMG-T relationship, are largely driven by differences in muscle mass and quality in young versus postmenopausal females. Additional studies are needed to more closely examine age-related differences in the EMG-T relationship, MUAP versus recruitment threshold relationship, and muscle mass to better understand the utility and limitations of these measurements.

Overall our data suggest that postmenopausal females exhibit significant reductions in muscle mass, muscle quality, and neuromuscular function compared to their younger female counterparts. Furthermore, our data suggest that although muscle quality is influential, muscle mass is likely a critical driver of the declines in neuromuscular function observed in these females, suggesting that the preservation of muscle mass should be a primary goal for females in the years leading up to, during, and immediately after menopause.

## Supplementary Material

421_2020_4532_MOESM1_ESM

## Figures and Tables

**Table 1 –:** Between group differences for the young versus postmenopausal females.

	Young n=30	Postmenopausal n=30	p value
	Mean ± SD	Mean ± SD	
Age (y)	20.7 ± 2.8	56.3 ± 4.7	<0.001*
Height (m)	163.8 ± 7.4	162.3 ± 6.5	0.407
Weight (kg)	70.3 ± 13.9	70.1 ± 10.3	0.94
Caloric Intake (kcal)	1503 ± 575.8	1560 ± 424.5	0.411
Protein Intake (g)	59.79 ± 26.22	67.45 ± 19.3	0.072
SMM (kg)	20.9 ± 3.0	18.6 ± 2.8	0.004*
FFMI (kg/m^2^)	16.6 ± 1.4	15.5 ± 1.3	0.002*
MVIT (Nm)	126.5 ± 33.7	109.4 ± 29.1	0.04*
PA (°)	5.4 ± 0.3	4.9 ± 0.4	<0.001*
mCSA (cm^2^)	19.8 ± 4.2	14.3 ± 3.9	<0.001*
EI (a.u.)	69.6 ± 10.07	79.5 ± 11.5	0.001*
MQ (cm^2^/a.u.)	0.3 ± 0.1	0.19 ± 0.07	<0.001*
Slope4o (mV·%MVIT^−1^)	0.0011 ± 0.0005	0.0008 ± 0.0005	0.009*
Intercept_40_ (mV)	0.0008 ± 0.0026	0.0021 ± 0.0017	0.03*
Slopeyo (mV·%MVTT^−1^)	0.0013 ± 0.0006	0.0010 ± 0.0006	0.005*
Intercept_70_ (mV)	0.0040 ± 0.0045	−0.0007 ± 0.0047	0.001*

SMM = skeletal muscle mass; FFMI = fat free mass index; MVIT = maximal voluntary isometric torque; PA = phase angle; mCSA = mean cross sectional area; EI = echo intensity; MQ = muscle quality;

* indicates a significant group difference between the young and postmenopausal females (p ≤ 0.05)

**Table 2 –:** Pearson’s partial correlation coefficients among the dependent variables after partialling out bodyweight.

	Height	SMM	FFMI	MVIT	PA	mCSA	EI	MQ	Slope_40_	Int_40_
**Height**	1									
**SMM**	0.660^†^	1								
**FFMI**	−0.444^†^	0.328*	1							
**MVIT**	0.240	0.522^†^	0.223	1						
**PA**	−0.191	0.521^†^	0.769^†^	0.410^†^	1					
**mCSA**	0.024	0.426^†^	0.416^†^	0.465^†^	0.549^†^	1				
**EI**	−0.087	−0.377^#^	−0.344^#^	−0.378^#^	−0.357^#^	−0.648^†^	1			
**MQ**	0.004	0.418^†^	0.452^†^	0.478^†^	0.514^†^	0.925^†^	−0.823^†^	1		
**Slope**_**40**_	0.225	0.465^†^	0.250	0.435^†^	0.298*	0.285*	−0.289*	0.325*	1	
**Intercept**_**40**_	−0.287*	−0.275*	−0.036	−0.007	0.032	−0.177	0.158	−0.224	−0.287*	1
**Slope**_**70**_	0.216	0.441^†^	0.227	0.410^†^	0.282*	0.307*	−0.291*	0.319*	0.915^†^	−0.255
**Intercept**_**70**_	−0.187	−0.258*	−0.088	−0.142	−0.067	−0.273	0.224	−0.250	−0.375^#^	0.561^†^

SMM = skeletal muscle mass; FFMI = fat free mass index; MVIC = maximal voluntary isometric contraction; PA = phase angle; mCSA = mean cross sectional area; EI = echo intensity; MQ = muscle quality;

* p ≤ 0.05;

# p≤ 0.01;

† p≤ 0.001

## ABBREVIATIONS:

ANCOVA: Analysis of Covariance
BIA: Bioelectrical impedance analysis
EI: Echo intensity
EMG: Electromyography
EMG-T: Electromyographic vs. Torque
FFMI: Fat free mass index
Intercept_40_; Intercept_70_: Intercept at 40%/70% maximal voluntary isometric torque
MVIC: Maximal voluntary isometric contraction
MVIT: Maximal voluntary isometric torque
MU: Motor Unit
MUAP: Motor Unit Action Potential Amplitude
mCSA: Muscle cross sectional area
MQ: Muscle quality
PA: Phase angle
SMM: Skeletal muscle mass
Slope_40_; Slope_70_: Slope at 40%/70% maximal voluntary isometric torque
sEMG: Surface electromyography
US: Ultrasound
VL: Vastus lateralis
