# Supplementary material for: Electromyographic amplitude versus torque relationships are different in young versus postmenopausal females and are related to muscle mass after controlling for bodyweight
Source: Eur J Appl Physiol. Author manuscript; Available in PMC 2022 Dec 19. (PMC9762459; doi:10.1007/s00421-020-04532-0)
Supplement: 421_2020_4532_MOESM1_ESM [file NIHMS1642523-supplement-421_2020_4532_MOESM1_ESM.docx]

**Supplementary Table 1** – Individual data for the slopes and intercepts for the EMG vs. Torque relationship at 40% and 70% maximal voluntary contraction in young and postmenopausal females.

|  |  | **Young Females** | | | | | |  | **Postmenopausal Females** | | | | | |
| --- | --- | --- | --- | --- | --- | --- | --- | --- | --- | --- | --- | --- | --- | --- |
| **Subject** |  | **Slope_40_** | **Int_40_** | **r** | **Slope_70_** | **Int_70_** | **r** |  | **Slope_40_** | **Int_40_** | **r** | **Slope_70_** | **Int_70_** | **r** |
| 1 |  | 0.0017 | -0.0023 | 0.9896 | 0.0015 | -0.0005 | 0.9785 |  | 0.0007 | 0.0017 | 0.9790 | 0.0008 | 0.0000 | 0.9877 |
| 2 |  | 0.0005 | 0.0005 | 0.9874 | 0.0007 | -0.0041 | 0.9680 |  | 0.0009 | 0.0023 | 0.9949 | 0.0009 | 0.0017 | 0.9945 |
| 3 |  | 0.0024 | -0.0031 | 0.9765 | 0.0023 | -0.0087 | 0.9768 |  | 0.0027 | 0.0019 | 0.9801 | 0.0031 | -0.0078 | 0.9750 |
| 4 |  | 0.0009 | 0.0008 | 0.9674 | 0.0009 | -0.0039 | 0.9842 |  | 0.0003 | 0.0016 | 0.9863 | 0.0005 | -0.0015 | 0.9865 |
| 5 |  | 0.0012 | -0.0024 | 0.9798 | 0.0010 | -0.0025 | 0.9886 |  | 0.0012 | -0.0004 | 0.9746 | 0.0013 | -0.0040 | 0.9666 |
| 6 |  | 0.0015 | 0.0023 | 0.9866 | 0.0017 | -0.0016 | 0.9922 |  | 0.0005 | 0.0007 | 0.9913 | 0.0005 | -0.0006 | 0.9876 |
| 7 |  | 0.0011 | 0.0032 | 0.9963 | 0.0012 | -0.0015 | 0.9819 |  | 0.0009 | -0.0016 | 0.9747 | 0.0010 | -0.0019 | 0.9889 |
| 8 |  | 0.0008 | -0.0005 | 0.9834 | 0.0008 | -0.0002 | 0.9936 |  | 0.0015 | 0.0008 | 0.9881 | 0.0013 | 0.0009 | 0.9886 |
| 9 |  | 0.0004 | 0.0003 | 0.9879 | 0.0007 | -0.0053 | 0.9586 |  | 0.0009 | 0.0027 | 0.9956 | 0.0010 | 0.0027 | 0.9937 |
| 10 |  | 0.0013 | 0.0042 | 0.9972 | 0.0015 | -0.0027 | 0.9801 |  | 0.0005 | 0.0048 | 0.9834 | 0.0010 | -0.0034 | 0.9785 |
| 11 |  | 0.0012 | 0.0044 | 0.9890 | 0.0012 | 0.0000 | 0.9934 |  | 0.0009 | 0.0033 | 0.9965 | 0.0008 | 0.0045 | 0.9841 |
| 12 |  | 0.0010 | -0.0016 | 0.9874 | 0.0012 | -0.0063 | 0.9666 |  | 0.0007 | -0.0011 | 0.9739 | 0.0010 | -0.0075 | 0.9578 |
| 13 |  | 0.0008 | 0.0006 | 0.9943 | 0.0010 | -0.0033 | 0.9875 |  | 0.0005 | 0.0005 | 0.9915 | 0.0007 | -0.0024 | 0.9768 |
| 14 |  | 0.0020 | -0.0063 | 0.9749 | 0.0023 | -0.0147 | 0.9749 |  | 0.0012 | 0.0058 | 0.9960 | 0.0011 | 0.0066 | 0.9911 |
| 15 |  | 0.0013 | -0.0013 | 0.9698 | 0.0018 | -0.0118 | 0.9696 |  | 0.0005 | 0.0038 | 0.9913 | 0.0008 | 0.0009 | 0.9894 |
| 16 |  | 0.0005 | 0.0014 | 0.9825 | 0.0009 | -0.0046 | 0.9741 |  | 0.0008 | 0.0013 | 0.9979 | 0.0009 | -0.0028 | 0.9829 |
| 17 |  | 0.0010 | 0.0007 | 0.9923 | 0.0009 | 0.0001 | 0.9761 |  | 0.0019 | 0.0033 | 0.9947 | 0.0021 | 0.0011 | 0.9882 |
| 18 |  | 0.0006 | 0.0030 | 0.9980 | 0.0007 | 0.0003 | 0.9699 |  | 0.0006 | 0.0023 | 0.9820 | 0.0005 | 0.0054 | 0.9497 |
| 19 |  | 0.0004 | 0.0014 | 0.9882 | 0.0007 | -0.0042 | 0.9694 |  | 0.0006 | 0.0027 | 0.9887 | 0.0006 | 0.0006 | 0.9887 |
| 20 |  | 0.0016 | 0.0049 | 0.9861 | 0.0020 | -0.0043 | 0.9643 |  | 0.0006 | 0.0030 | 0.9606 | 0.0007 | 0.0021 | 0.9290 |
| 21 |  | 0.0010 | 0.0000 | 0.9924 | 0.0014 | -0.0076 | 0.9513 |  | 0.0004 | 0.0033 | 0.9547 | 0.0005 | 0.0010 | 0.9837 |
| 22 |  | 0.0015 | 0.0029 | 0.9972 | 0.0015 | 0.0017 | 0.9918 |  | 0.0006 | 0.0023 | 0.9907 | 0.0007 | -0.0023 | 0.9806 |
| 23 |  | 0.0011 | -0.0003 | 0.9816 | 0.0013 | -0.0043 | 0.9872 |  | 0.0008 | 0.0008 | 0.9748 | 0.0008 | -0.0027 | 0.9785 |
| 24 |  | 0.0010 | -0.0020 | 0.9347 | 0.0010 | -0.0033 | 0.9734 |  | 0.0005 | 0.0018 | 0.9808 | 0.0007 | -0.0006 | 0.9873 |
| 25 |  | 0.0006 | 0.0017 | 0.9807 | 0.0009 | -0.0012 | 0.9883 |  | 0.0008 | 0.0053 | 0.9927 | 0.0009 | 0.0012 | 0.9815 |
| 26 |  | 0.0005 | 0.0011 | 0.9932 | 0.0008 | -0.0043 | 0.9601 |  | 0.0011 | 0.0018 | 0.9406 | 0.0023 | -0.0190 | 0.9634 |
| 27 |  | 0.0007 | 0.0031 | 0.9966 | 0.0008 | 0.0022 | 0.9883 |  | 0.0008 | 0.0027 | 0.9913 | 0.0009 | 0.0024 | 0.9909 |
| 28 |  | 0.0008 | 0.0040 | 0.9789 | 0.0009 | 0.0017 | 0.9818 |  | 0.0005 | 0.0022 | 0.9920 | 0.0006 | 0.0005 | 0.9739 |
| 29 |  | 0.0021 | 0.0025 | 0.9843 | 0.0023 | -0.0089 | 0.9735 |  | 0.0004 | 0.0006 | 0.9911 | 0.0004 | 0.0009 | 0.9900 |
| 30 |  | 0.0017 | -0.0004 | 0.9925 | 0.0024 | -0.0157 | 0.9567 |  | 0.0007 | 0.0024 | 0.9933 | 0.0008 | 0.0024 | 0.9950 |
| **Mean** |  | 0.0011 | 0.0008 | 0.9849 | 0.0013 | -0.0040 | 0.9767 |  | 0.0008 | 0.0021 | 0.9841 | 0.0010 | -0.0007 | 0.9803 |
| **SD** |  | 0.0005 | 0.0026 | 0.0124 | 0.0006 | 0.0045 | 0.0117 |  | 0.0005 | 0.0017 | 0.0133 | 0.0006 | 0.0047 | 0.0146 |

Int_40/70_: Intercept at 40%/70% maximal voluntary contraction; Slope_40/70_: Slope at 40%/70% maximal voluntary contraction
